# Over-Reduced State of Mitochondria as a Trigger of “β-Oxidation Shuttle” in Cancer Cells

**DOI:** 10.3390/cancers14040871

**Published:** 2022-02-10

**Authors:** Zhivko Zhelev, Akira Sumiyoshi, Ichio Aoki, Dessislava Lazarova, Tatyana Vlaykova, Tatsuya Higashi, Rumiana Bakalova

**Affiliations:** 1 Department of Molecular Imaging and Theranostics, National Institutes for Quantum Science and Technology (QST), Chiba 263-8555, Japan; zh_zhelev@yahoo.com (Z.Z.); sumiyoshi.akira@qst.go.jp (A.S.); aoki.ichio@qst.go.jp (I.A.); higashi.tatsuya@qst.go.jp (T.H.); 2 Faculty of Medicine, Trakia University, 6000 Stara Zagora, Bulgaria; tatyana.vlaykova@trakia-uni.bg; 3 Institute of Biophysics and Biomedical Engineering, Bulgarian Academy of Sciences, 1113 Sofia, Bulgaria; 4 Faculty of Medicine, Sofia University “St. Kliment Ohridski”, 1407 Sofia, Bulgaria; dessislaval@yahoo.com

**Keywords:** cancer, metabolism, mitochondrial fatty acid oxidation, β-oxidation shuttle

## Abstract

**Simple Summary:**

This review proposes the idea that many peculiarities of the cancer cell metabolism are easier to explain considering the incomplete combustion of fatty acids in the cancerous mitochondria due to their over-reduced redox state. Recent studies indicate that overactivated mitochondrial β-oxidation may significantly alter the mitochondrial redox state and vice versa. Thus, the impaired redox state of cancerous mitochondria can ensure the continuous operation of β-oxidation by disconnecting it from the Krebs cycle and connecting it to the citrate–malate shuttle. This could create a new metabolic state/pathway in cancer cells, which we have called the “β-oxidation shuttle”. This artificial pathway is inefficient as an energy source. However, when combined with acetyl-CoA consuming pathways, such as fatty acid synthesis and mevalonate pathways, it is a source of cataplerosis, leading to biomass accumulation, accelerated oxygen consumption, and, ultimately, a source of proliferation.

**Abstract:**

A considerable amount of data have accumulated in the last decade on the pronounced mitochondrial fatty acid oxidation (mFAO) in many types of cancer cells. As a result, mFAO was found to coexist with abnormally activated fatty acid synthesis (FAS) and the mevalonate pathway. Recent studies have demonstrated that overactivated mitochondrial β-oxidation may aggravate the impaired mitochondrial redox state and vice versa. Furthermore, the impaired redox state of cancerous mitochondria can ensure the continuous operation of β-oxidation by disconnecting it from the Krebs cycle and connecting it to the citrate–malate shuttle. This could create a new metabolic state/pathway in cancer cells, which we have called the “β-oxidation-citrate–malate shuttle”, or “β-oxidation shuttle” for short, which forces them to proliferate. The calculation of the phosphate/oxygen ratio indicates that it is inefficient as an energy source and must consume significantly more oxygen per mole of ATP produced when combined with acetyl-CoA consuming pathways, such as the FAS and mevalonate pathways. The “β-oxidation shuttle” is an unconventional mFAO, a separate metabolic pathway that has not yet been explored as a source of energy, as well as a source of cataplerosis, leading to biomass accumulation, accelerated oxygen consumption, and, ultimately, a source of proliferation. The role of the “β-oxidation shuttle” and its contribution to redox-altered cancer metabolism provides a new direction for the development of future anticancer strategies. This may represent the metabolic “secret” of cancer underlying hypoxia and genomic instability.

## 1. Over-Reduced State of Mitochondria and Activation of Fatty Acid Oxidation in Cancer

Until recently, discussions on the cancer cell metabolism have mainly focused on glycolysis, as well as on genetic defects found in the Krebs cycle and mitochondria in general. In the last few years, there has been growing interest in the role of mitoepigenetic regulation in cancer cells [1]. Recent studies have demonstrated that mitochondrial fatty acid oxidation (mFAO) is vital for many types of cancer cells, and they cannot exist without this metabolic pathway [2,3,4,5]. This review proposes the idea that many “mysteries” about the peculiarities of cancer cell metabolism, including respiration and even hypoxia, seem easier to explain in the light of the incomplete combustion of fatty acids in cancerous mitochondria. Studies indicate that overactivated mitochondrial β-oxidation may aggravate the impaired mitochondrial redox state and vice versa, whereby the impaired redox state of cancerous mitochondria can ensure the continuous operation of β-oxidation by disconnecting it from the Krebs cycle and connecting it to the citrate–malate shuttle. This could create a new metabolic state/pathway in cancer cells, which we have called the “β-oxidation-citrate–malate shuttle”, or “β-oxidation shuttle” for short. This pathway can force them to proliferate. This abnormal, unconventional metabolic state has not yet been described. 

This concept is based on the following findings:

### 1.1. mFAO Is Activated in Many Types of Cancer Cells

Over the last decade, mFAO has been found to be activated under conditions common to solid tumors and cancer cells. For example, the loss of expression or activity of acetyl-CoA carboxylase 2 (ACC2), as well as overexpression of carnitine palmitoyl transferase 1 (CPT1), have been observed under acidic conditions (as in cancer) or environments enriched with fatty acids [6]. ACC2 and CPT1 are key regulatory enzymes of mitochondrial β-oxidation (Figure S1 in the Supplementary Materials). The mechanisms of mFAO expression in cancer cells suggest that this pathway could be overactivated depending on the energy needs of the cell. The evidence for this is provided below.

It has been reported that fatty acid synthesis (FAS) is elevated in glioma cells, but the same is true for mFAO [7,8,9]. Indeed, mFAO is the best source for production of acetyl-CoA, which is the main precursor of FAS together with NADPH. FAS and mFAO inhibitors have been shown to suppress the proliferation of glioma cells [10]. Moreover, some cancer cells prefer to be located close to adipocytes, the main store of fats [11]. In 2011, Nieman et al. found that co-culturing ovarian cancer cells with human-derived adipocytes resulted in increased β-oxidation by activating CPT1 and acyl-CoA oxidase 1 [12]. The rate-limiting enzyme of FAS (fatty acid synthase) has been found to be overexpressed in ovarian cancer, whose inhibitors cause growth arrest and apoptosis in the same type of cells [13].

Numerous studies have demonstrated the role of irregularly activated mFAO in the metabolism and survival of cancer cells [14,15,16,17]. In 2013 and 2014, acidosis was reported to lead to a decrease in glucose consumption by cancer cells and an increase in the anaplerotic pathways of the Krebs cycle [18,19]. In this case, the activation of mFAO could be suggested because of decreased glucose consumption. This is a well-known regulation associated with the “Randle cycle”. In 2014, Schlaepfer et al. demonstrated that the CPT1 inhibitor suppressed the proliferation of prostate cancer cells and tumor growth in mice [20]. The authors found that CPT1-knockdown in these cells causes inhibition of their proliferation, which is accompanied by decreased oxidation of palmitate. In 2016, Corbet et al. discovered that mFAO could be irregularly activated in acidosis-adapted cancer cells via the deacetylation of histones, which leads to a decreased expression of ACC2 [6]. The authors also observed a significant non-enzymatic acetylation of mitochondrial proteins, caused by elevated levels of acetyl-CoA. In turn, this leads to the acetylation and inactivation of complex I in the mitochondrial electron transport chain (ETC). This relationship between acidification and β-oxidation was known as the “Corbet–Feron effect” [10], which hints at its universality. Recently, Luis et al. reported that, under certain conditions, such as obesity-mimicking status, cancer cells can spend lipids and amino acids to synthesize glucose from lactate via gluconeogenesis [21]. The authors have called this phenomenon “Warburg effect inversion”. They cultured breast cancer cells in three different media: high glucose concentration, low glucose concentration, and high concentration of fatty acids released by adipocytes. Medium containing adipocytes significantly increases the viability and proliferation of breast cancer cells, as well as their migration rate and aggression. Cancer cells in a high-fat environment excrete significant amounts of glucose, pyruvate, and acetate. Here, it is important to note that a distinctive feature of activated mFAO (in this case) is the increased consumption of lactate and glutamine.

Another mechanism for the overactivation of mFAO in cancer cells is through the modulation of ACC2 activity by hydroxylation [22]. Prolyl hydroxylase 3 has been found to hydroxylate and activate ACC2, whose malonyl-CoA product inhibits mFAO. Prolyl hydroxylase 3 is an oxygen sensor, known as the deactivator of hypoxia inducible factor 1-alpha (HIF-1α) [23]. This enzyme is essential for the regulation of hypoxia. In the presence of oxygen, prolyl hydroxylate 3 hydroxylates HIF-1α and deactivates it by directing it to ubiquitination. The finding that prolyl hydroxylase 3 also hydroxylates and activates the metabolic enzyme ACC2 means that, at normal oxygen levels, mFAO should be inhibited. However, in acute myeloid leukemia cells, the level of prolyl hydroxylase 3 has been found to be low and thus potentiates their dependence on mFAO in normoxia [22]. Other mechanisms for downregulating ACC2 have also been described [24].

The studies mentioned above demonstrate that mFAO can be overactivated by suppressing ACC2 under acidic conditions, decreasing the activity of prolyl hydroxylase 3 in hypoxia, or by increasing the availability of fatty acids, which are very important factors in cancer metabolism. This mechanism appears to be universal given that the acidic environment of cancer cells and hypoxia are common features of tumors and can activate mFAO.

At present, it is difficult to predict the number of cancers that are dependent on mFAO. Most likely, these are cancers that have been shown to be dependent on oxidative phosphorylation (OXPHOS). Amoedo et al. identified two subgroups of lung carcinomas: high and low OXPHOS expressing tumors [25]. High OXPHOS expressing tumors poorly incorporated [^18^F]fluorodeoxy-glucose and had an increased expression of the mitochondrial trifunctional fatty acid oxidation enzyme (TFP), particularly TFP subunit alpha, compared to the paired adjacent tissue. The genetic and pharmacological inhibition of the TFP subunit alpha affects tumor growth in vivo. Trimetazidine, an approved drug inhibitor of TFP used in cardiology, disrupts the interaction between TFP and complex I of the ETC, leading to a cellular redox and energy crisis [26].

Mitochondrial FAO is considered an important factor in the growth of cancer cells, as well as an important target in the development of new drugs for various cancers, such as pancreatic, prostate, leukemia, lymphoma, and ovarian [11,15,25,27,28,29].

Although studies on mFAO do not cover many different types of cancer, publications on this phenomenon have increased enormously over the last decade, and mFAO inhibitors have been successful in treating cancer cells [30,31,32,33,34].

Accumulating evidence of overactivated mFAO in cancer cells, in addition to abnormally activated metabolic pathways, such as FAS and pentose phosphate pathway (PPP), suggest the existence of a metabolic cycle in which FAS coexists with mFAO. This cycle is prohibited during normal cellular metabolism under normal conditions.

### 1.2. Mitochondrial β-Oxidation May Inhibit Pyruvate Combustion and Complex I via Acetylation of Mitochondrial Proteins

It is well known that β-oxidation leads to the accumulation of high concentrations of acetyl-CoA (Figure S1 in the Supplementary Materials). Thus, the abnormal expression of β-oxidation in cells activates the non-enzymatic acetylation of mitochondrial proteins. In many pathologies, including cancer, this acetylation can lead to severe mitochondrial dysfunction if mitochondrial sirtuins (SIRT3, SIRT4, and SIRT5) are not expressed and activated simultaneously. This acetylated type of mitochondrial dysfunction is characterized by the minimal conversion of pyruvate to acetyl-CoA and decreased activity of complex I.

In terms of the evidence for these assumptions, over the last decade, a high-fat diet has been shown to decrease SIRT3 expression, impair mitochondrial function, and decrease reliance on glycolytic substrates [35,36,37,38,39]. Furthermore, in 2007, Koves et al. found that obesity-related insulin resistance and a high-fat diet are characterized by excessive β-oxidation in skeletal muscles and impaired transition to a carbohydrate substrate during the fasting-to-diet transition [37]. The authors reported that factors inhibiting the import of fatty acids in mitochondria protect against lipid-induced insulin resistance.

In 2011, Choudhury et al. subjected mice to a chronic high-fat diet and observed decreased SIRT3 activity in the liver and a three-fold decrease in hepatic NAD^+^ levels [35]. The authors identified 193 proteins that were preferably acetylated in mice on a high-fat diet compared to controls on a normal diet. SIRT3-deficient mice have been found to show even greater hyperacetylation of gluconeogenic and mitochondrial proteins in a high-fat diet. In addition to increased acetylation, SIRT3-deficient mice exhibited disruption of mitochondrial complexes II, III, and V.

In 2015, Lantier et al. demonstrated that muscles in SIRT3-deficient mice exhibit profound mitochondrial dysfunction with decreased reliance on glycolytic substrates and increased reliance on fatty acid substrates [36]. The authors observed that respiration decreases in the muscle fibers of SIRT3-deficient mice when malate-glutamate substrate is used, while oxygen consumption is significantly higher with malate palmitoyl-carnitine substrate. These studies are consistent with the idea that fatty acid catabolism could antagonize glucose catabolism by acetylating mitochondrial proteins. This is a further development of Randle’s postulates [40], who stated that the provision of lipid fuel promotes β-oxidation and suppresses glycolysis and oxidation of pyruvate due to inhibition of hexokinase, phosphofructokinase, and pyruvate dehydrogenase (PDH). Decreased SIRT3 expression when fatty acids are high in availability may cause a permanent dependence of cells on fatty acids as a fuel and compromise the transition of their metabolism to glucose combustion.

Recently, it has also been reported that the antagonism between fatty acid catabolism and glucose catabolism in a high-fat diet does not depend only on decreased SIRT3 expression and/or activity [37,38,39]. The acylcarnitine system has been found to be involved in the acetylation of mitochondrial proteins and in the antagonism of fatty acids versus glucose. Koves et al. emphasized that acylcarnitine may play a role in insulin resistance, and acylcarnitine production is considered a detoxifying system that allows mitochondrial efflux of excess acyl-groups [37]. This suggestion is based on their own data and Ramsay’s study [37,38]. Ramsay proposed that the role of the carnitine system is to maintain homeostasis in the acyl-CoA pools of the cell, keeping the acyl-CoA/CoA pool constant even under conditions of very high acyl-CoA turnover [38]. Davies et al. demonstrated that carnitine acetyltransferase (CrAT) could be responsible for elevated levels of acetyl-CoA and the acetylation of mitochondrial proteins, which is associated with metabolic dysfunction [39]. CrAT is an enzyme that buffers the mitochondrial pool of acetyl-CoA by converting short-chain acyl-CoAs to their membrane-permeable acylcarnitine analogues. CrAT-deficiency increases tissue acetyl-CoA levels and susceptibility to diet-induced lysine acetylation of broad-spectrum mitochondrial proteins, which is accompanied by decreased whole-body glucose control [39]. CrAT was found to be responsible for the export of excess acetyl-CoA from the mitochondrial matrix, and CrAT-knockout mice (CrAT^−^/^−^) showed a similar over-acetylated phenotype as SIRT3-knockout mice (SIRT3^−^/^−^).

### 1.3. Over-Reduced State of Mitochondria Can Inhibit the Krebs Cycle but Not the “β-Oxidation”

Under hypoxic conditions, as well as under rest conditions, the NADH/NAD^+^ ratio in mitochondria increases to maximal levels [41,42,43]. At high NADH levels, the process of mitochondrial β-oxidation cannot be easily suppressed based on the principle of feedback. In contrast, the enzymes of the Krebs cycle, α-ketoglutarate dehydrogenase (α-KGDH) and isocitrate dehydrogenase 3 (IDH3), are very sensitive to NADH and are inhibited by these reducing equivalents not only in hypoxia but also when a cell contains high amounts of ATP [44,45]. Evidence for these statements is briefly described below.

When pyruvate is combusted to CO_2_ and H_2_O in the Krebs cycle, there are no substantial end products of the overall reaction that can inhibit this process other than ATP. This is a prerequisite for mitochondrial ATP to directly inhibit pyruvate combustion by inhibiting respiration. It should be noted that, in this case, the cell relies entirely on mitochondrial ATP as a feedback mechanism, and this is the only factor that controls the Krebs cycle and the assimilation of pyruvate. The feedback regulation of glucose metabolism by ATP involves inhibition of the Krebs cycle by NADH, which is not consumed by complex I. We propose that elevated levels of ATP and NADH do not have the ability (power) to inhibit mFAO itself, specifically the part of mFAO preceding the Krebs cycle—β-oxidation. This hypothesis is based on the studies described below.

The regulation of mitochondrial β-oxidation is entrusted mainly to AMP-activated protein kinase (AMPK) and the mitochondrial ACC2—AMPK-ACC2 mechanism (Figure S1 in the Supplementary Materials). This mechanism should stop mFAO at normal and high glucose concentrations, respectively, and normal and high ATP levels when AMPK is deactivated (Figure S1A in the Supplementary Materials). However, the AMPK-ACC2 mechanism should activate the mFAO when glucose was depleted, ATP/ADP ratio decreased, APM appeared, and AMPK was activated (Figure S1B in the Supplementary Materials). Therefore, we assume that, if the AMPK-ACC2 mechanism is compromised, mitochondrial β-oxidation can get out of control and be functional even when: (i) the cell contains a large amount of ATP, (ii) the mitochondrial matrix contains a high amount of NADH, and (iii) the Krebs cycle is inhibited in certain segments.

The complete oxidation of one molecule of saturated acyl-CoA, such as palmitoyl-CoA, must go through a series of chain-length specific enzymes, including acyl-CoA dehydrogenases (ACADs), enoyl-CoA hydratases (EHs), 3-hydroxyacyl-CoA dehydrogenases (3HADs), and 3-ketothiolases (KATs), to catalyze the cyclic release of acetyl-CoA units [46]. Only two of the four reactions are oxidative. The reactions catalyzed by FAD-dependent ACADs cannot be directly inhibited by ATP, high transmembrane potential, or high levels of FAD, which is a prosthetic group in these enzymes. No ATP binding sites have been reported for these enzymes. They are not proton pumps and are independent of the transmembrane potential.

The 3HAD enzymes that catalyze the second step of β-oxidation are NAD^+^-dependent, and, because NAD^+^ and NADH are cofactors, their concentrations may affect their enzymatic activity. In 1998, Eaton et al. investigated the sensitivity of isolated mitochondrial TFP activity to different concentrations of NADH and acetyl-CoA, two end-products of β-oxidation [47]. The authors found that the TFP was relatively insensitive to the NAD^+^/NADH ratio. The inhibition of its enzyme activity begins at an NAD^+^/NADH ratio of less than 1 or 2.5 (when NADH increases above 50 to 60%). Sahlin and Katz demonstrated that mitochondrial NADH in skeletal muscles at rest does not exceed 60%, while, in active heart muscle, it does not exceed 13% [42]. Therefore, it can be concluded that the TFP cannot be inhibited at physiological concentrations of NADH. In comparison, α-KGDH, a key enzyme in the Krebs cycle, is almost completely inhibited by 50% of NADH and its activity decreases more than two-fold at 25% of NADH [48,49]. The inhibition of NAD-dependent IDH is even more pronounced [50]. The IC_50_ values for different isoforms of NAD-dependent IDH vary between 7.8% and 9.4% of NADH versus NAD^+^ and reach almost complete inhibition at 35 to 40% of NADH [50].

**Figure 1:**
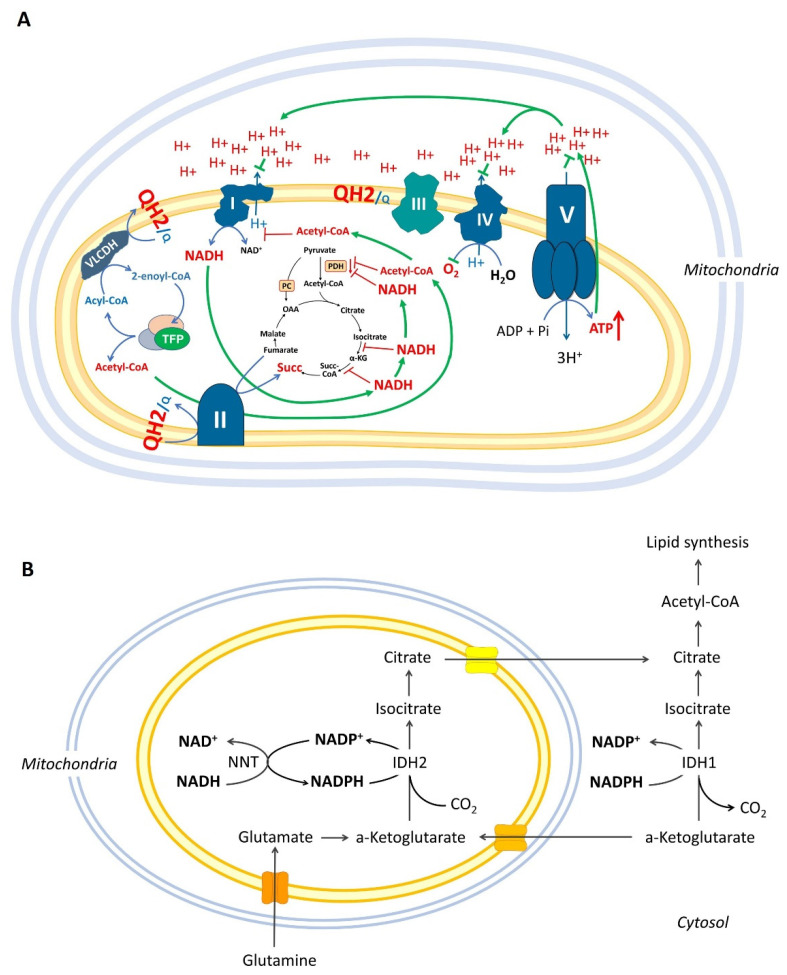
(**A**) Possible consequences of the overactivated β-oxidation in mitochondria: high Q10H_2_/Q10 ratio and accumulation of acetyl-CoA and succinate. This could lead to high levels of NADH and acetyl-CoA that inhibit the Krebs cycle, PDH, and complex I of the mitochondrial ETC. The green arrows indicate accumulated metabolites. The red blunt ends indicate the inhibition of a particular enzyme. (**B**) NNT-catalyzed conversion of NADH to NADPH in mitochondria and reductive carboxylation of α-ketoglutarate to isocitrate, catalyzed by IDH1 and IDH2. Abbreviations: ETC: electron transport chain; α-KG: α-ketoglutarate; IDH: isocitrate dehydrogenase; NNT: NAD(P) transhydrogenase; OAA: oxaloacetate; Q: Coenzyme Q10 (oxidized form); QH_2_: coenzyme Q10 (reduced form); PDH: pyruvate dehydrogenase; PC: pyruvate carboxylase; Succ: succinate; Succ-CoA: succinyl-CoA; TFP: trifunctional protein; VLCDH: very long chain acyl CoA dehydrogenase.

If the concept that ATP and NADH do not have enough power to inhibit the spiral process of mitochondrial β-oxidation is right, the result from its overactivation will be an accumulation of four main products: reduced coenzyme Q10 (Q10H_2_), succinate, more NADH, and acetyl-CoA, as reported by Randle, as well as in more recent studies (Figure 1A) [31,51,52]. High levels of NADH and/or acetyl-CoA will inhibit the Krebs cycle, PDH, and complex I.

Recently, Guarás et al. found that the shift from glucose to fatty acid metabolism increases electron flux through FAD-dependent enzymes, which saturates the Q-pool and leads to reverse electron transport (RET) through complex I [52]. In addition, this is accompanied by the downregulation of complex I. It is not clear whether this downregulation occurs through the direct destruction of complex I by increased production of reactive oxygen species (ROS) or by ROS-mediated regulatory pathways. Their results support the idea that the coenzyme Q10 redox state acts as a metabolic sensor that fine-tunes the mitochondrial ETC configuration to adjust the electron flux to the FADH_2_/NADH ratio.

The excretion of oncometabolite succinate from cancer cells is considered an important signal that stimulates their migration, invasion, and metastases [53,54,55]. Many dysregulations of succinate dehydrogenase (SDH) in cancer cells are discussed [53].

Recently, Li et al. reported that the stimulation of cardiomyocytes with palmitate increased mFAO, which led to the intracellular accumulation of succinate and its release in the extracellular space [56]. Interestingly, the authors found that accumulated succinate induces a hypoxia-like condition via increased HIF-1α expression, as well as impaired PDH activity via the upregulation of pyruvate dehydrogenase kinase 4 (PDK4) expression. It should be noted that increased succinate has also been found in the blood of humans and mice with obesity [57,58]. It is well known that, in obese individuals, the level of free fatty acids in the blood is markedly high [59]. In turn, this provides strong support of a relationship between overactivated β-oxidation and SDH inhibition.

### 1.4. Reductive Carboxylation, Over-Reduced State of Mitochondrial Matrix, and Krebs Cycle Impairments

In cancer cells, overactivated β-oxidation, characterized by a compromised AMPK-ACC2 mechanism, could be combined with acetylated mitochondria, hypoxia, and/or impaired ETC function. The combination of these factors could further increase the pressure of NADH in the mitochondrial matrix. NADH, which is not consumed by complex I, is oxidized and converted to NADPH by mitochondrial nicotinamide nucleotide transhydrogenase (NNT) to increase the reductive carboxylation of α-ketoglutarate (α-KG) (Figure 1B). The reductive carboxylation of α-KG was found to be activated in many types of cancer cells, and the driving force is the reduced state of the matrix in the form of NAD(P)H [60,61,62]. At such a redox state, the Krebs cycle is at least partially inhibited. This raises the question: is it possible for β-oxidation to work at an inhibited Krebs cycle?

Defects in complex I and complex III directly support reductive carboxylation in the normal direction of the Krebs cycle. While Gameiro et al. noted that the source(s) of NADPH contribution to reductive carboxylation in mitochondria is/are unknown, the authors also found that the NNT enzyme is an important intermediate of the driving force that causes reductive glutaminolysis [60]. Another study demonstrated that cancer cells with defective mitochondria use glutamine-dependent reductive carboxylation rather than oxidative metabolism as the major pathway for citrate formation [61]. The authors found that the reductive glutamine-dependent pathway is the dominant mode in the metabolism of cancer cells (renal carcinoma) derived from patients with mutations in fumarate hydratase, as well as in cells with normal mitochondria subjected to acute pharmacological inhibition of ETC [61]. They assumed that the cause and driving force of this mechanism is the increased NADH/NAD^+^ ratio in the mitochondrial matrix because of the decreased oxidative capacity of the Krebs cycle. According to their results, the NADH/NAD^+^ ratio could be partially dissipated by NAD(P)-transhydrogenase, which transfers reducing equivalents from NADH to NADPH and, in turn, can cause NADPH-dependent reductive carboxylation by isocitrate dehydrogenases (IDH1 and IDH2) (Figure 1B). In hypoxic melanoma cells, the reverse flux of the Krebs cycle through IDH1 and IDH2 has been found to also be required for lipogenesis and viability [63]. Although this is a good model to explain the mechanism of reductive carboxylation, it is incomplete. It is worth noting that the inhibition of ETC may increase the NADH/NAD^+^ ratio for a short time and may decrease the oxidative capacity of the Krebs cycle. This means that the Krebs cycle is inhibited and can no longer produce NADH. Therefore, other processes that can produce NADH will need to be examined.

Another decisive factor in this regulation is defects in the Krebs cycle. Such defects are common in cancer cells. Mutations have been found in several key enzymes of the Krebs cycle, including IDH, fumarate hydratase (FH), and SDH [53,61,62,64]. Furthermore, α-KGDH is known to be downregulated in some cancers [45,65]. Recently, the citrate–malate shuttle was found overexpressed in cancer cells with an impaired Krebs cycle [66]. Although the activity of mFAO has not been studied in cancer cells containing simultaneously mutations in the Krebs cycle, it is worth asking: how can mFAO work with a disrupted Krebs cycle due to mutations in SDH, FH, and/or a-KGDH? In fact, the β-oxidation pathway, in combination with such impairments in the Krebs cycle, may work well together with the citrate–malate shuttle. Such an unconventional metabolic pathway can be used as an energy source, which is an alternative to glycolysis and standard mFAO. When acetyl-CoA cannot be completely oxidized, citrate leaves the mitochondria, causing enormous cataplerosis. The energy efficiency of such a metabolic pathway has not yet been considered compared to other cellular energy sources, but it is quite unique, as described below.

## 2. Definition of the “β-Oxidation Shuttle” and Its Components: Energy Efficiency

**Figure 2:**
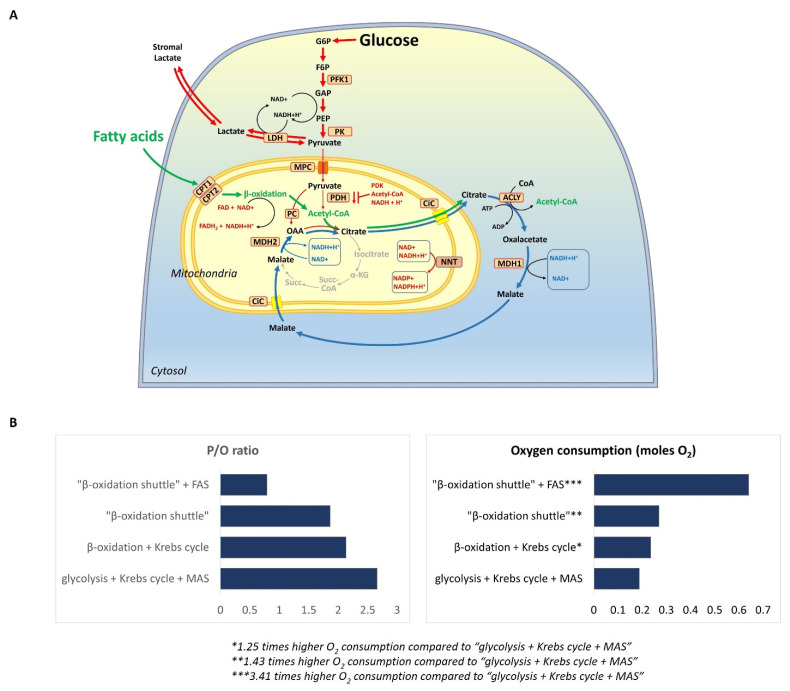
(**A**) Schematic representation of the “β-oxidation shuttle” in mitochondria: the relation between β-oxidation, the citrate–malate shuttle, and the formation of a separate and independent metabolic pathway, which has its own energy efficiency, own oxygen consumption, and own influence on other metabolic pathways. The green arrows indicate the metabolic flux from fatty acids. The red arrows indicate the metabolic flux from glucose. The red blunt ends indicate the inhibition of a particular enzyme. The blue arrows indicate the citrate-malate shuttle. (**B**) Phosphate/oxygen (P/O) ratio and oxygen consumption in the combustion of glucose and palmitate in the “β-oxidation shuttle” and other metabolic pathways—a comparison. Abbreviations: ACLY: ATP citrate lyase; CIC: mitochondrial citrate carrier; CTP1 and CTP2: carnitine palmitoyl transferases 1 and 2; F6P: fructose-6-phosphate/fructose-1,6-bisphosphate; G6P: glucose-6-phosphate; GAP: glyceraldehyde-3-phosphate; α-KG: α-ketoglutarate; MAS: malate–aspartate shuttle; MPC: mitochondrial pyruvate carrier; NNT: NAD(P) transhydrogenase; OAA: oxaloacetate; PDK: pyruvate dehydrogenase kinase; PDH: pyruvate dehydrogenase; PC: pyruvate carboxylase; PFK1: phosphofructokinase-1; PK: pyruvate kinase; PEP: phosphoenolpyruvate; Succ: succinate.

When β-oxidation is overactivated due to an impaired AMPK-ACC2 mechanism or the high availability of fatty acids, it may change the redox state of mitochondria in favor of an increased NADH/NAD^+^ ratio because the mitochondrial trifunctional protein is less sensitive to inhibition by an NADH-dependent feedback mechanism [47]. If the ATP demand of the cell is low, it will further alter the reduced state of the mitochondrial matrix and increase NADH. Effects such as ETC dysfunction and partial hypoxia, capable of changing the redox balance of the mitochondrial matrix in the direction of a high NADH/NAD^+^ ratio, could also be combined with overactivated mitochondrial β-oxidation and could inhibit certain enzymes of the Krebs cycle. Mutations in some enzymes of the Krebs cycle increase the ability of β-oxidation to function without part of the Krebs cycle. If β-oxidation is not connected to the Krebs cycle but rather to the citrate–malate shuttle, it forms a separate and independent metabolic pathway, which has its own energy efficiency, oxygen consumption, and impact on other metabolic pathways. We denoted this metabolic state “β-oxidation shuttle” to distinguish it from the other metabolic states, as well as from the standard mitochondrial β-oxidation working with the functional Krebs cycle (Figure 2A). The “β-oxidation shuttle” brings a major advantage to the cell related to the export of citrate and NADPH from the mitochondria into the cytoplasm and supports all anabolic processes (Figure S2 in the Supplementary Materials).

The “β-oxidation shuttle” is able to maintain the NADH/NAD^+^ ratio at the highest possible level in the mitochondrial matrix. The dissipation of this reduced state occurs by transferring reducing equivalents from NADH to NADP^+^ via NNT and by exporting reducing equivalents, such as NADPH, from the mitochondrial matrix to the cytoplasm. NNT is located on the inner mitochondrial membrane and is an energy-dependent enzyme. It is driven by the mitochondrial proton motive force (Δ*p*), and its activity is directly dependent on the respiratory state of the mitochondria [67,68].

The significance of NNT overexpression in cancer progression has been described in several studies [69,70,71]. Lactate dehydrogenase (LDH), which produces cytoplasmic NAD^+^, is able to dissipate this pressure by decreasing the import of cytoplasmic reducing equivalents into the matrix via the malate–aspartate shuttle [72]. Thus, activating LDH in cancer cells that are addicted to the “β-oxidation shuttle” should act as a compensatory mechanism to decrease the irregularly changed redox state of the mitochondrial matrix. Pyruvate can be eliminated from the metabolism by other reactions, such as transamination into alanine, if pyruvate is not required for ATP production in the mitochondria. Alanine could be easily excreted by cells and be used by the liver in the glucose–alanine cycle. However, cancer cells prefer to remove excess lactate through LDH and face a low pH. We assume that the goal of increased anaerobic glycolysis in the cell over the need for pyruvate in the mitochondria is not only to produce ATP but also to compensate the redox state of the mitochondrial matrix through LDH expression. Some types of cancer cells also produce additional pyruvate through the malic enzyme [73]. This may also be a compensatory mechanism for decreasing the NADH/NAD^+^ ratio.

We propose that the “β-oxidation shuttle” consists of mitochondrial β-oxidation and a citrate–malate shuttle. In turn, the citrate–malate shuttle consists of a transmembrane transporter, mitochondrial citrate/isocitrate carrier (CIC), and several enzymes, including ATP-citrate lyase (ACLY) and malate dehydrogenases 1 and 2 (MDH1 and MDH2) (Figure 2A). What is the impact of these components in the context of carcinogenesis?

Citrate is at the crossroads of many metabolic pathways and is an indispensable source of carbons in the mitochondria and cytosol, a key substrate for the generation of energy, as well as an allosteric modulator of several enzymes (Figures S1 and S2 in the Supplementary Materials). When glucose is abundant, the majority of intracellular citrate comes predominantly from the oxidative decarboxylation of pyruvate in the mitochondria. It is then exported to the cytoplasm via the CIC in exchange for malate. CIC plays an important role in lipogenesis and is also a key component of the isocitrate–oxaloacetate and citrate–malate shuttles. Numerous studies have demonstrated the pro-inflammatory and pro-oncogenic effects of CIC, as well as its participation in reversing the Warburg effect [74,75,76,77,78]. The fundamental role of CIC and its upregulation in cancer, inflammation, and beyond are well known. High levels of CIC in tumors are thought to allow for adaptation to nutritional stress and resistance to mitochondrial respiration injury [75]. It is interesting to note that CIC expression is also regulated by diet. For example, starvation and a diet enriched with polyunsaturated fatty acid could significantly downregulate the expression of this transporter protein [79]. Decreased expression of proteins involved in FAS, including CIC, is also relevant to the loss of adipose tissue mass in cancer-bearing animals subjected to chemotherapy [80].

ACLY is a key metabolic enzyme that catalyzes the conversion of citrate to acetyl-CoA in the cytoplasm. ACLY is upregulated in cancer cells and is required for their growth [81]. ACLY overexpression has been associated with increased tumor progression in many cancers, including breast, lung, brain, colorectal, hepatocellular, and others [82,83,84,85,86,87,88]. ACLY expression and inhibition have also been associated with other chronic diseases, such as diabetes, obesity, non-alcoholic fatty liver disease, cardiovascular diseases, inflammatory disorders, and neurodegenerative diseases [81].

An important circumstance related to the “β-oxidation shuttle”, which has not been discussed so far, is its dependence on mitochondrial MDH2. MDH2 is part of the Krebs cycle, and the catalyzed reaction is referred to as reversible, although its standard Gibbs free energy is positive, and the backward reaction is preferred under standard conditions [89]. However, MDH2 is allosterically regulated by three metabolites: citrate, malate, and oxaloacetate [90]. These three metabolites inhibit MDH2 activity in the backward direction from NADH to NAD^+^. They may also inhibit MDH2 activity in the forward direction from NAD^+^ to NADH, but only at low concentrations of malate and NAD^+^ as substrates. In contrast, citrate, malate, and oxaloacetate activate MDH2 in the forward direction from NAD^+^ to NADH at higher substrate concentrations. It was noted above that the mitochondrial NADH in skeletal muscles at rest is between 36% and 60% [42]. Therefore, it can be concluded that NAD^+^ in the mitochondrial matrix should be sufficient to prevent the inhibition of the forward reaction. However, the presence of malate and citrate in the matrix should activate the enzyme in the forward direction.

This model of regulation of the activity of MDH2 also suggests that higher amounts of enzyme in the mitochondria will accelerate the forward reaction (in the direction of NADH) and will support the existence of the “β-oxidation shuttle” in cancer cells. Recently, the level of MDH2 in urine was found to be higher in patients with non-small-cell lung cancer compared to the same parameter in the healthy population [91]. The same study reported that MDH2 knockdown in lung cancer cell lines inhibits cell proliferation.

Hanse et al. demonstrated that cytosolic MDH1 can activate glycolysis by producing NAD^+^ in the cytoplasm as an alternative to LDH as a supplier of NAD^+^ in various cancer cell lines [92]. The amplification of MDH1 occurs with an impressive frequency in human tumors and correlates with a poor prognosis. On the other hand, if additional processes producing NADH in the cytoplasm are activated in cancer cells, they will increase the NADH/NAD^+^ ratio and MDH1 will decrease the additional reductive pressure in the cytoplasm. Cytosolic aldehyde dehydrogenases (ALDHs) and the polyol pathway for the synthesis of fructose from glucose have also been discussed as providers of NADH in the cytoplasm and have been found to be abnormally activated in cancer (Figure S3 in the Supplementary Materials) [93,94,95,96]. Both factors (ALDHs and polyol pathway) appear to be important for cancer cell survival. Thus, additional suppliers of the cytoplasm with reducing equivalents, such as NADH, could be interpreted as accelerators of MDH1 for malate production and for increasing the availability of malate in mitochondria, which could activate mitochondrial MDH2.

Interesting results have recently been published by Sullivan et al. [97]. They observed that changing the redox state using ETC inhibitors and other redox alterations may affect the synthesis of aspartate. Cancer cells, whose growth was suppressed by ETC inhibitors, died from aspartate deficiency (not from ATP deficiency) and could be saved by the addition of aspartate [97]. The second finding is that ETC inhibitors could inhibit the malate–aspartate shuttle and increase the cytoplasmic NADH/NAD^+^ ratio. This can lead to the inhibition of glycolysis and ATP depletion. Although this study showed that mitochondrial function is important for the survival of cancer cells, this does not mean that the production of mitochondrial ATP is the only vital factor. MDH2 and cytoplasmic MDH1 are part of the citrate–malate shuttle, but they are also part of the malate–aspartate shuttle [72]. The malate–aspartate shuttle transfers reducing equivalents from the cytoplasm to the mitochondria and is also involved in the pathways for aspartate synthesis.

Our hypothesis is that the “β-oxidation shuttle” is expressed when the redox state of the matrix reaches the point of inhibition of α-KGDH and NAD-dependent IDH (or at least NAD-dependent IDH only), but MDH2 is not inhibited. A further increase in the NADH/NAD^+^ ratio may inhibit MDH2, which will simultaneously stop three processes: the malate–aspartate shuttle, the synthesis of aspartate, and the citrate–malate shuttle. With regard to which of these three processes is the most important for the survival of cancer cells, surprisingly, it seems that this is not the provision of reducing equivalents for ATP synthesis but the synthesis of aspartate [97]. Cancer cells require many biochemical processes to grow and survive. Two of them are essential and are provided by mitochondria, namely (i) the synthesis of citrate, which is a precursor for the synthesis of lipid ingredients (phospholipids, sphingolipids, coenzyme Q, and cholesterol), and (ii) the synthesis of aspartate, which is a precursor for the synthesis of purines, pyrimidines, and, ultimately, DNA.

Partial defects in mitochondrial complexes I and III should exert pressure on the Q10H_2_/Q10 ratio and increase the inhibitory effect of β-oxidation on SDH. The inhibition of ETC will also increase the NADH/NAD^+^ ratio to inhibit α-KGDH and NAD-dependent IDH (Figure 1). In fact, some mutations in the subunits of complex I that are responsible for its deficient (altered) activity lead to the stimulation of tumor growth and metastasis [65]. However, further inhibition of ETC could cause problems, even for cancer cells that are completely addicted to glycolysis and do not rely on mitochondria for ATP synthesis [65]. Although the “β-oxidation shuttle” can provide ATP to the cell, the main function of this metabolic pathway seems to provide cytoplasmic citrate for lipid synthesis and to support aspartate synthesis and DNA synthesis, respectively, which is crucial for the survival of cancer cells [97].

Assuming that the “β-oxidation shuttle” is a key characteristic of some types of cancer cells, we should be able to compare its energy efficiency with that of glycolysis when combined with the combustion of pyruvate in the Krebs cycle. The energy efficiency and oxygen consumption of the “β-oxidation shuttle” can be calculated by following the generally accepted opinions and approaches for calculating the phosphate/oxygen (P/O) ratio from NADH and FADH_2_ [98]. 

The comparison between the “β-oxidation shuttle” with the standard “β-oxidation + Krebs cycle” shows that the partial combustion of palmitate in the “β-oxidation shuttle” produces 26 moles of ATP, while its combustion in the “β-oxidation + Krebs cycle” produces 98 moles of ATP from 1 mole of palmitate (Figure 2B). Therefore, 3.76 times less ATP is produced in the “β-oxidation shuttle” compared to the “β-oxidation + Krebs cycle”. A comparison between the “β-oxidation shuttle” and the standard glucose combustion in the “glycolysis + Krebs cycle + malate–aspartate shuttle” shows that the oxygen consumption per mole of ATP is 0.269 moles of oxygen in palmitate partial oxidation versus 0.1875 in glucose oxidation, which is approximately 1.43 times higher oxygen consumption in the “β-oxidation shuttle”. The “β-oxidation shuttle” shows the lowest P/O ratio of 1.86. Moreover, in the cycle “β-oxidation shuttle + FAS”, when NADPH is coming only from PPP, the oxygen consumption is 0.64 moles per mole of ATP. Therefore, “β-oxidation shuttle + FAS” consumes approximately 3.4 times more oxygen per mole of ATP than “glycolysis + Krebs cycle + malate–aspartate shuttle”.

We need to clarify that these calculations are approximate and need to be refined because of the existence of the final product in each metabolic pathway, such as acetyl-CoA used in fatty acid synthesis. To calculate the actual energy efficiency and the actual oxygen consumption in a metabolic pathway that has a final product, the energy required to utilize this product must also be included in the equations. However, this did not significantly change the differences between the four combinations of metabolic pathways described in Figure 2B.

Theoretically, we can consider the coexistence of the two processes, “β-oxidation shuttle + FAS”, as a separate metabolic cycle when β-oxidation is overactivated and the Krebs cycle is inhibited (even partially). This artificial process is energetically possible and relies only on NADPH availability. This NADPH could be produced in PPP or reductive glutaminolysis if combined with NADPH-dependent IDH and isocitrate-dependent NADPH exporting shuttle.

The ability of cancer cells to express both metabolic pathways simultaneously, namely FAS and mFAO, seems strange. We are accustomed to assuming that it is not energetically profitable for the cell to synthesize a substance and decompose it simultaneously. Typically, much more energy is used for synthesis than is released during the decomposition of the same compound. However, this rule does not apply when energy comes from NADPH. The cycle “β-oxidation shuttle + FAS” is energetically beneficial to some extent because β-oxidation of palmitate produces more ATP compared to the amount of ATP spent on its synthesis. The difference in energy is covered by NADPH, which can be produced in several ways. Thus, the artificial cycle “β-oxidation shuttle + FAS” could be considered as a process for converting NADPH energy into energy of some amount of ATP.

## 3. Conclusions

In this article, we did not discuss whether the “β-oxidation shuttle” really exists or in how many cancer cells it is expressed. Our goal was to use this model to calculate the energy efficiency and oxygen consumption in irregularly activated mitochondrial β-oxidation when the Krebs cycle is inhibited and the final product acetyl-CoA is used in the synthesis of fatty acids.

There are several reasons why this cycle may not occur. For example, the lipotoxicity of palmitate and stearate could be an important factor. This may explain why some cancer cells are addicted to extrinsic fats. However, this does not prevent the coexistence of the two metabolic pathways, FAS and “β-oxidation shuttle”. Acetyl-CoA produced in the “β-oxidation shuttle” must be consumed, and the cost should be paid. This cost is the energy consumed for the utilization of acetyl-CoA, which could be the energy for FAS. In 2020, it was reported that inhibitors of fatty acid binding proteins, a family of proteins that transport fatty acids across the membranes, suppressed tumor growth by regulating fatty acid metabolism [99]. This suggests that cancer cells appear to be more dependent on external fatty acids than on their own.

It should be noted that the “β-oxidation shuttle” fits well with the Warburg effect, which has not yet been convincingly explained. When cancer cells are removed from their natural hypoxic environment, they may have normal oxygen consumption, but they still prefer to convert glucose anaerobically. The increased oxygen demand of the “β-oxidation shuttle”, combined with the inhibited activity of PDH and partially inhibited β-oxidation enzymes, can lead to seemingly normal oxygen consumption combined with lactate production, inefficient mitochondrial ATP synthesis, and huge cataplerosis. This explains the Warburg effect, as well as uncontrolled growth and proliferation. In this context, glycolysis and OXPHOS should not be antagonized because OXPHOS can coexist with anaerobic glycolysis when the “β-oxidation shuttle” is expressed. The possible inefficient synthesis of ATP in the “β-oxidation shuttle” provides conditions for some tumors to be dependent simultaneously on OXPHOS and glycolysis (such as gliomas) [7,8,9]. The synthesis of mitochondrial ATP becomes more inefficient in relation to the “β-oxidation shuttle” as more cancer cells should depend on glycolysis.

The main role of the “β-oxidation shuttle” should be the export of citrate and huge cataplerosis, the main characteristic of cancer cells. On the other hand, we consider that the over-reduced mitochondrial matrix is a major cause of the expression of β-oxidation outside the Krebs cycle, and, at the same time, it is a consequence of overactivated mitochondrial β-oxidation. LDH helps cancer cells to decrease the reduced state of the mitochondrial matrix by producing NAD^+^. This explains anaerobic glycolysis as a compensatory mechanism not only for ATP production but also for mitigating the consequences of the enormously increased redox state of the mitochondria.

The calculations show that the “β-oxidation shuttle” is inefficient as an energy source and must consume at least two to four times more oxygen per mole of ATP produced when combined with acetyl-CoA consuming pathways, such as FAS and the mevalonate pathway (Figure 2B). This could be a clue to a new potential source of hypoxia in cancer.

The “β-oxidation shuttle” could be expressed to some extent under control in non-cancerous proliferating cells, as well as in certain types of immune cells and embryonic cells. However, this expression should be reversible, whereas, in cancer cells, it should be irreversible. The “β-oxidation shuttle” may be tightly connected with some chronic diseases, such as diabetes 2, obesity, fatty liver disease, and cardiac hypertrophy, but it is difficult to predict whether this “weird” metabolic pathway is reversible in these diseases. These assumptions require experimental validation, and whether it is possible to restore the normal functionality of mitochondria after they fall into this metabolic dysfunction will need to be clarified.

Thus, the role of the “β-oxidation shuttle” in impaired cancer metabolism should be investigated in future studies. This artificial cycle may hold the key to elucidating the metabolic mechanism by which cancer contributes to hypoxia and genomic instability.

## Supplementary Materials

The following supporting information can be downloaded at: https://www.mdpi.com/article/10.3390/cancers14040871/s1, Figure S1: Regulation of FAS and mFAO by AMPK at normal or high ATP/ADP ratio or low ATP/ADP ratio: AMPK-ACC2 mechanism; Figure S2: Links between the “β-oxidation shuttle”, malate–aspartate shuttle, and glutaminolysis; Figure S3: Polyol pathway in solving the redox equation of the “β-oxidation shuttle”.

## Abbreviations

ACAD	acyl-CoA dehydrogenase
ACC1	acetyl-CoA carboxylase
ACLY	ATP-dependent citrate lyase
ALDH	aldehyde dehydrogenase
AMPK	adenosine monophosphate-activated protein kinase
CIC	citrate/isocitrate carrier
CPT	carnitine palmitoyl transferase
CrAT	carnitine acetyltransferase
EH	enoyl-CoA hydratase
ETC	electron transport chain
mFAO	mitochondrial fatty acid oxidation
FAS	fatty acid synthesis
FH	fumarate hydratase
3HAD	3-hydroxyacyl-CoA dehydrogenase
HIF-1α	hypoxia inducible factor one alpha
IDH	isocitrate dehydrogenase
KAT	3-ketothiolases
α-KG	alpha-ketoglutarate
α-KGDH	alpha-ketoglutarate dehydrogenase
LDH	lactate dehydrogenase
MCD	malonyl-CoA decarboxylase
MDH	malate dehydrogenase
NNT	nicotinamide nucleotide transhydrogenase
OXPHOS	oxidative phosphorylation
PDH	pyruvate dehydrogenase
PDK	pyruvate dehydrogenase kinase
PPP	pentose phosphate pathway
Q10 and Q10H_2_	coenzyme Q10 (oxidized and reduced forms)
RET	reverse electron transport
ROS	reactive oxygen species
SDH	succinate dehydrogenase
SIRT	sirtuin
TFP	mitochondrial trifunctional fatty acid oxidation enzyme
