# Supplementary material for: Over-Reduced State of Mitochondria as a Trigger of “β-Oxidation Shuttle” in Cancer Cells
Source: Cancers (Basel). 2022 Feb 10;14(4):871. doi: 10.3390/cancers14040871 (PMC8870273; doi:10.3390/cancers14040871)
Supplement: Supplementary file 1 [file cancers-14-00871-s001.zip › cancers-1525421-supplementary.pdf]

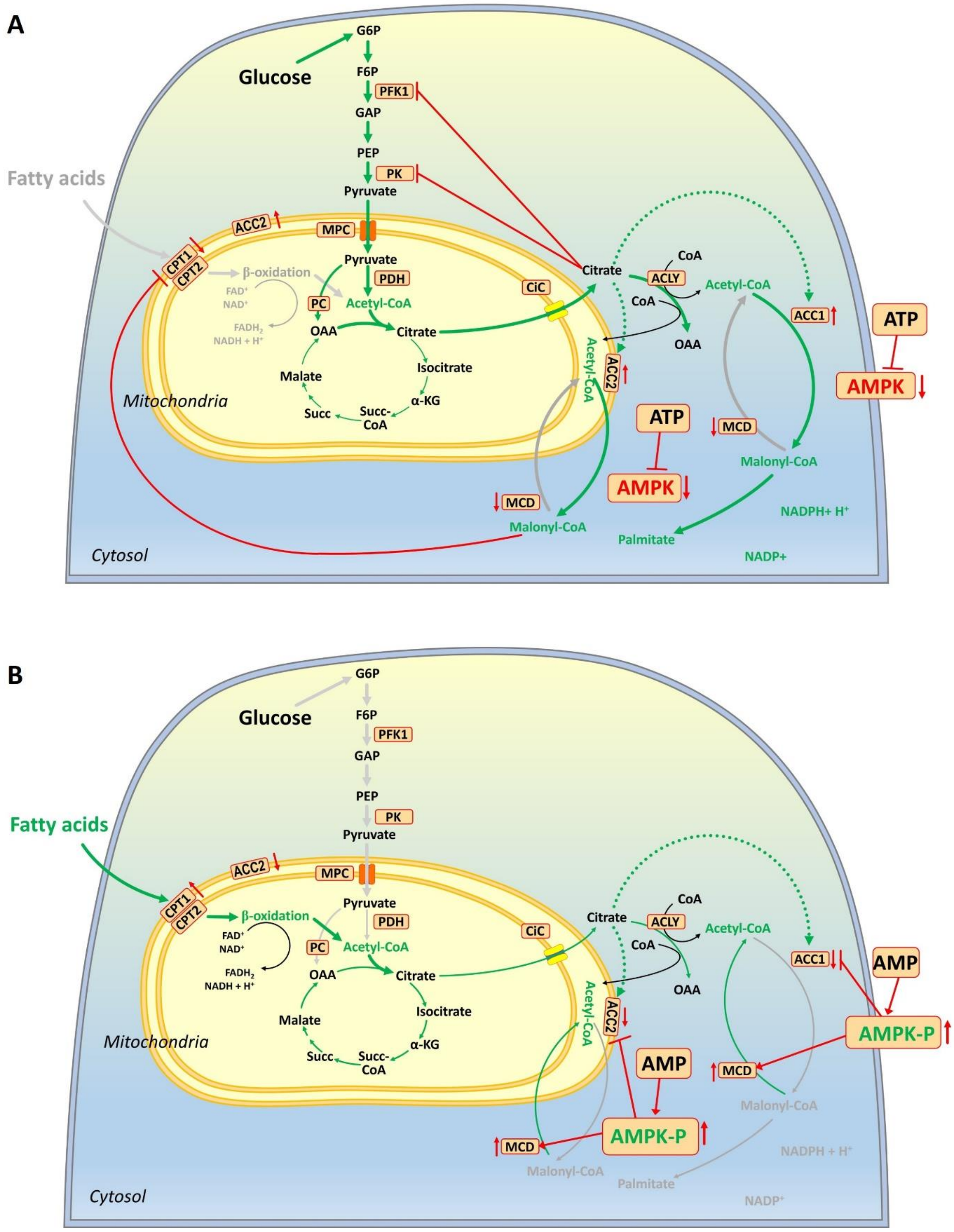

Figure S1. Regulation of FAS and mFAO by AMPK at normal or high ATP/ADP ratio **(A)** or low ATP/ADP ratio **(B)**. The green arrows indicate the expressed/activated pathway. The grey arrows indicate the suppressed/deactivated pathway. The red blunt ends indicate the inhibition of a particular enzyme. The red arrows indicated the activation of a particular enzyme. Abbreviations: ACC1 and ACC2: acetyl-CoA carboxylases 1 and 2; ACLY: ATP citrate lyase; AMPK: 5′AMP-activated protein kinase (dephosphorylated state); CIC: mitochondrial citrate carrier; CTP1 and CTP2: carnitine palmitoyl transferases 1 and 2; F6P: fructose-6-phosphate/fructose-1,6-bisphosphate; G6P: glucose-6-phosphate; GAP: glyceraldehyde-3-phosphate; α-KG: α-ketoglutarate; MCD: malonyl-CoA decarboxylase; MPC: mitochondrial pyruvate carrier; OAA: oxaloacetate; PDH: pyruvate dehydrogenase; PC: pyruvate carboxylase; PFK1: phosphofructokinase-1; PEP: phosphoenolpyruvate; PK: pyruvate kinase; Succ: succinate. Briefly, the ATP/ADP ratio is the most important energy indicator

in cells, and AMPK is the main sensor of this ratio, as well as a modulator of the activities of ACC1 and ACC2. At normal concentrations and normal or high ATP/ADP ratios, AMPK is deactivated and ACC1 and ACC2 are found in their active dephosphorylated and polymer forms (Figure 1A) [1,2]. ACC1 and ACC2 are involved in the synthesis of malonyl-CoA, which inhibits the key regulatory enzyme of mFAO, CPT1, and thus inhibits the β-oxidation of fatty acids in mitochondria [3,4]. The metabolite citrate appears in the cytoplasm. Citrate is an inhibitor of two glycolytic enzymes (PFK1 and PK), which is a feedback mechanism that stops the overcharging of cells with energy and precursors for synthetic processes. Citrate inhibits glycolysis only if its concentration exceeds the capacity of ACLY. ACLY is primarily regulated at the genetic level and its activity does not appear to depend on any metabolite other than citrate [5]. Citrate is also an activator of ACC1 and ACC2. This may induce the depletion of citrate in the cytoplasm by its conversion to acetyl-CoA, which in turn is converted to malonyl-CoA and is involved in FAS. Thus, all excess glucose is directed to its conversion into fatty acids. Malonyl-CoA should be considered primarily as a trigger of FAS, a precursor of FAS, and an inhibitor of mFAO [6]. The existence of the enzyme malonyl-CoA decarboxylase (MCD) seems useless in the case of active glucose degradation but makes sense in the case of a rapid change in the direction of glucose deficiency. When glucose is depleted and the ATP/ADP ratio decreases, AMP appears because of the function of adenylate kinase. AMPK was activated, and ACC1 and ACC2 were inactivated (Figure 1B). In this case, malonyl-CoA should be removed by activated MCD to rapidly eliminate the inhibition of CPT1 and to activate mFAO. This activates mitochondrial β-oxidation and stops the loss of energy from fatty acid re-synthesis. The subsequent recovery of the normal ATP/ADP ratio should return the cell to its normal state by deactivating AMPK, activating ACC1 and ACC2, and deactivating MCD. This restores the normal dependence of the cell on glucose, if available.

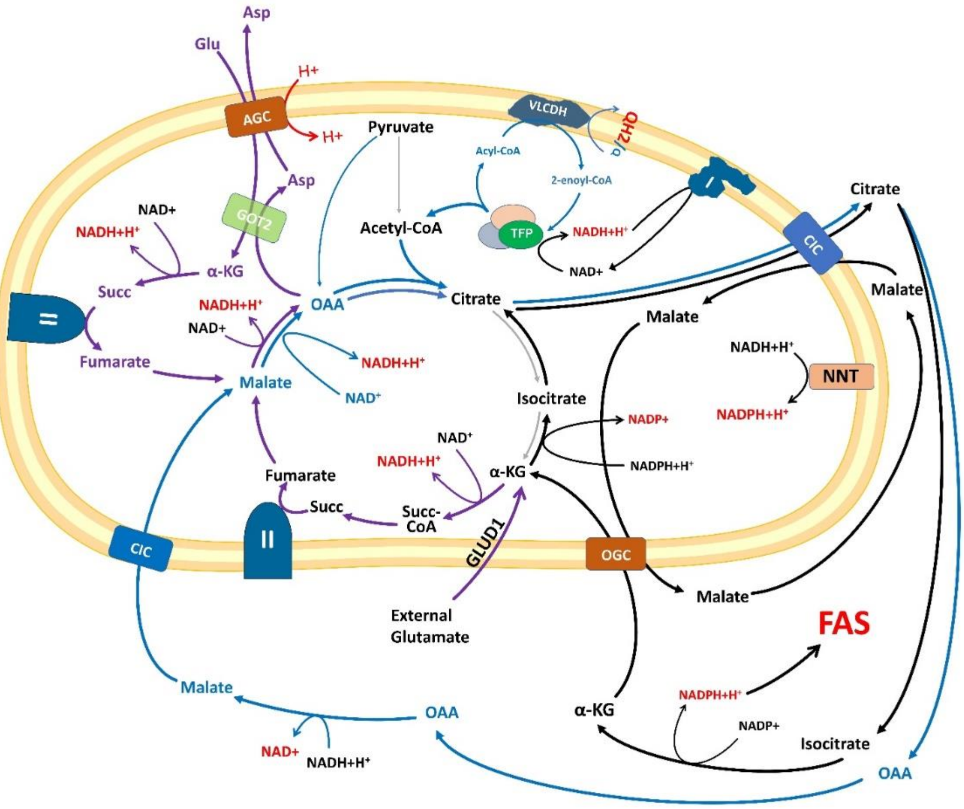

Figure S2. Links between the "β-oxidation shuttle", the malate-aspartate shuttle, and glutaminolysis pathways. The blue arrows indicate the "β-oxidation shuttle". The purple arrows indicate oxidative glutaminolysis and aspartate synthesis pathways. The black arrows indicate citrate-isocitrate shuttle. Legend: AGC – aspartate-glutamate carrier; Asp – aspartate; CIC – mitochondrial citrate carrier; FAS – fatty acid synthesis; Glu – glutamate; GLUD1 – glutamate dehydrogenase 1; GOT2 – glutamic-oxaloacetic transaminase 2; a-KG – a-ketoglutarate; NNT – NAD(P) transhydrogenase; OAA – oxaloacetate; OGC – oxoglutarate carrier; Succ – succinate; TFP – trifunctional protein; VLCDH – very long chain dehydrogenase.

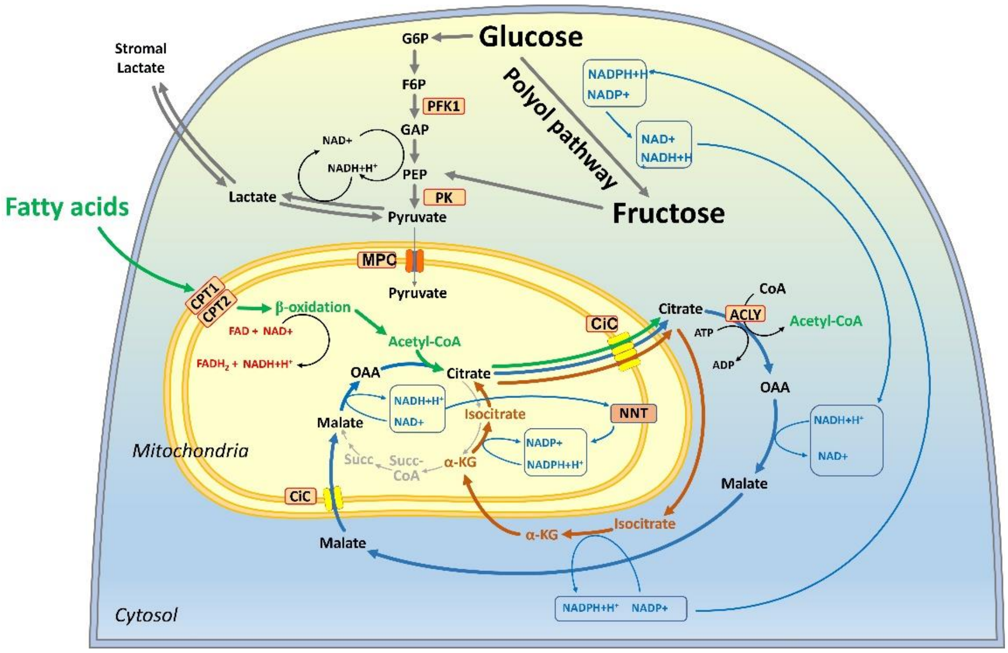

Figure S3. Polyol pathway in solving the redox equation of the "β-oxidation shuttle". The green arrows indicate the metabolic flux from fatty acids. The red arrows indicate the metabolic flux from glucose. The blue arrows indicate the malate-citrate shuttle. The brown arrows indicate the citrate-isocitrate shuttle. Abbreviations: ACLY: ATP citrate lyase; CIC: mitochondrial citrate carrier; CTP1 and CTP2: carnitine palmitoyl transferases 1 and 2; F6P: fructose-6-phosphate/fructose-1,6-bisphosphate; G6P: glucose-6-phosphate; GAP: glyceraldehyde-3-phosphate; α-KG: α-ketoglutarate; MPC: mitochondrial pyruvate carrier; NNT: NAD(P) transhydrogenase; OAA: oxaloacetate; PC: pyruvate carboxylase; PFK1: phosphofructokinase-1; PK: pyruvate kinase; PEP: phosphoenolpyruvate; Succ: succinate.

References

1. Hardie, D.G.; Alessi, D.R. LKB1 and AMPK and the cancer-metabolism link - ten years after. BMC Biol. **2013**, *11*, 36. doi: 10.1186/1741-7007-11-36.
2. Thampy, K.G.; Wakil, S.J. Regulation of acetyl-coenzyme A carboxylase. II. Effect of fasting and refeeding on the activity, phosphate content, and aggregation state of the enzyme. J Biol Chem. **1988**, *263*, 6454–6458. PMID: 2896194.
3. Abu-Elheiga, L.; Almarza-Ortega, D.B.; Baldini, A.; Wakil, S.J. Human acetyl-CoA carboxylase 2. Molecular cloning, characterization, chromosomal mapping, and evidence for two isoforms. J Biol Chem. **1997**, *272*, 10669–10677. doi: 10.1074/jbc.272.16.10669.
4. Abu-Elheiga, L.; Brinkley, W.R.; Zhong, L.; Chirala, S.S.; Woldegiorgis, G.; Wakil, S.J. The subcellular localization of acetyl-CoA carboxylase 2. Proc Natl Acad Sci U S A. **2000**, *97*, 1444–1449. doi: 10.1073/pnas.97.4.1444.
5. Khwairakpam, A.D.; Banik, K.; Girisa, S.; Shabnam, B.; Shakibaei, M.; Fan, L.; Arfuso, F.; Monisha, J.; Wang, H.; Mao, X.; Sethi, G.; Kunnumakkara, A.B. The vital role of ATP citrate lyase in chronic diseases. J Mol Med (Berl). **2020**, *98*, 71–95. doi: 10.1007/s00109-019-01863-0.
6. McGarry, J.D.; Mannaerts, G.P.; Foster, D.W. A possible role for malonyl-CoA in the regulation of hepatic fatty acid oxidation and ketogenesis. J Clin Invest. **1977**, *60*, 265–270. doi: 10.1172/JCI108764.
